# Deep Learning-Based Differentiation of Vertebral Body Lesions on Magnetic Resonance Imaging

**DOI:** 10.3390/diagnostics15151862

**Published:** 2025-07-24

**Authors:** Hüseyin Er, Murat Tören, Berkutay Asan, Esat Kaba, Mehmet Beyazal

**Affiliations:** 1 Faculty of Medicine, Recep Tayyip Erdoğan University, Training and Research Hospital, Rize 53020, Türkiye; huseyinertrbzn@gmail.com (H.E.); esatkaba04@gmail.com (E.K.); 2 Department of Electrical and Electronics Engineering, Recep Tayyip Erdogan University, Rize 53020, Türkiye; murat.toren@erdogan.edu.tr (M.T.); berkutay_asan21@erdogan.edu.tr (B.A.)

**Keywords:** metastasis, vertebral body lesions, MRI, deep learning, YOLO-v8, detection, classification

## Abstract

**Objectives:** Spinal diseases are commonly encountered health problems with a wide spectrum. In addition to degenerative changes, other common spinal pathologies include metastases and compression fractures. Benign tumors like hemangiomas and infections such as spondylodiscitis are also frequently observed. Although magnetic resonance imaging (MRI) is considered the gold standard in diagnostic imaging, the morphological similarities of lesions can pose significant challenges in differential diagnoses. In recent years, the use of artificial intelligence applications in medical imaging has become increasingly widespread. In this study, we aim to detect and classify vertebral body lesions using the YOLO-v8 (You Only Look Once, version 8) deep learning architecture. **Materials and Methods:** This study included MRI data from 235 patients with vertebral body lesions. The dataset comprised sagittal T1- and T2-weighted sequences. The diagnostic categories consisted of acute compression fractures, metastases, hemangiomas, atypical hemangiomas, and spondylodiscitis. For automated detection and classification of vertebral lesions, the YOLOv8 deep learning model was employed. Following image standardization and data augmentation, a total of 4179 images were generated. The dataset was randomly split into training (80%) and validation (20%) subsets. Additionally, an independent test set was constructed using MRI images from 54 patients who were not included in the training or validation phases to evaluate the model’s performance. **Results:** In the test, the YOLOv8 model achieved classification accuracies of 0.84 and 0.85 for T1- and T2-weighted MRI sequences, respectively. Among the diagnostic categories, spondylodiscitis had the highest accuracy in the T1 dataset (0.94), while acute compression fractures were most accurately detected in the T2 dataset (0.93). Hemangiomas exhibited the lowest classification accuracy in both modalities (0.73). The F1 scores were calculated as 0.83 for T1-weighted and 0.82 for T2-weighted sequences at optimal confidence thresholds. The model’s mean average precision (mAP) 0.5 values were 0.82 for T1 and 0.86 for T2 datasets, indicating high precision in lesion detection. **Conclusions:** The YOLO-v8 deep learning model we used demonstrates effective performance in distinguishing vertebral body metastases from different groups of benign pathologies.

## 1. Introduction

The prevalence of malignancies and other disease groups in the spine has significantly increased with aging and the extension of life expectancy in modern times [1]. Direct radiography, computed tomography (CT), and magnetic resonance imaging (MRI) are the primary imaging methods used in the diagnosis of spinal diseases [2]. Due to its high soft tissue resolution, ability to better evaluate the anatomical location and extent of lesions, and early detection of bone marrow changes, MRI is the gold standard imaging method for spinal diseases [3,4]. Metastases, benign tumors, infections, and compression fractures are common lesions in the spine. Although MRI has high diagnostic accuracy, differences in interpretation among physicians and the morphological similarities of lesions can pose challenges in differential diagnoses [5,6]. Pathological tissue biopsy may be necessary for a definitive diagnosis; however, as an invasive procedure, it carries a risk of complications [7]. Even if a biopsy is performed, it may not always lead to a definitive diagnosis [8]. Therefore, the development and improvement of noninvasive diagnostic methods are of great importance. Recently, deep learning applications have become widely used in medical imaging. 

In this study, the YOLO-v8 deep learning model was used for the detection and classification of vertebral body lesions. The aim is to develop a model that provides high accuracy in differential diagnosis and to demonstrate the potential of deep learning algorithms in this field. Additionally, the study aims to obtain results that will make significant contributions to clinical diagnostic processes.

## 2. Materials and Methods

This study is a non-randomized, observational, cross-sectional, and uncontrolled research conducted by retrospectively reviewing the imaging archive of patients in our hospital. We confirm that all procedures carried out in the study comply with ethical standards and follow the 1964 Helsinki Declaration. 

### 2.1. MRI Protocols

Patients who underwent thoracic and lumbar spinal MRI scans using a 1.5 T MRI (Siemens Magnetom Area, Erlangen, Germany) device in our hospital between January 2019 and March 2024 were included in our study. Patients were excluded if they had inadequate image quality, no pathological findings on MRI, a history of spinal instrumentation, lesions smaller than 1 cm, metastases without confirmatory PET-CT imaging, or a history of radiotherapy. The images consist of sagittal plane T1- and T2-weighted Turbo Spin Echo (TSE) sequences without fat suppression. Sequential sagittal image slices, in which partial volume effects were minimized and lesions were most clearly visualized, were used for analysis. Each image was evaluated independently, and no 3D volumetric information was included in the analysis process. A total of 392 lesions from 235 patients were included in the study.

### 2.2. Image Preprocessing

All images of the patients included in the study were evaluated by a radiologist with 15 years of experience, and diagnostic groups were created for acute compression fractures, metastases, hemangiomas, atypical hemangiomas, and spondylodiscitis. Images obtained in Digital Imaging and Communications in Medicine (DICOM) format from the Picture Archiving and Communication System (PACS) system were exported in JPEG format due to their lower file size and faster processing advantages. During this conversion, contrast optimization and windowing techniques were applied to preserve image quality as much as possible. During the image processing phase of the study, raw images with a resolution of 1403 × 937 pixels were converted into standardized images of 640 × 640 pixels using specific focal slices to enhance consistency during analysis and optimize processing time. The resolution of 640 × 640 was chosen primarily because it is the default input size for the YOLOv8 architecture and allows for sufficient representation of smaller lesions. After the images were prepared at 640 × 640 resolution, the model was trained on a 32-core CPU infrastructure. As a result of this process, a feature map with dimensions of 640 × 640 × 32 was obtained for each input image. Finally, the regions of interest (ROI), manually identified by the radiologist to fully encompass the lesion and vertebral corpus on sagittal MRI, were annotated with labels. This process generated the final images to be used for object detection training.

### 2.3. Augmentation

**Figure 1:**
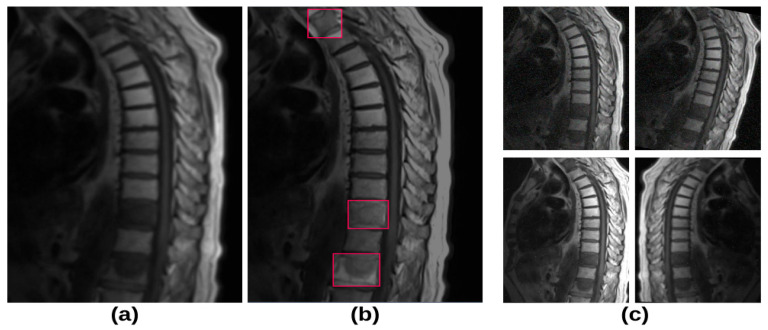
Dataset preparation: (**a**) T1-weighted MRI image of the spine, (**b**) classification and labeling of groups by the radiologist (red box), (**c**) image augmentation using PIL, including cropping (0–20% zoom), rotation (−15° to 15°), shearing (±15° horizontally and ±15° vertically), noise addition, and brightness adjustment.

In this study, data augmentation techniques were applied to prevent overfitting due to the relatively small sample size and the unequal distribution of lesion groups. Using Python’s version 3.9. PIL (Python Imaging Library, PyhtonWare, Linköping, Sweden), the following augmentations were performed: horizontal flipping, cropping (0–20% zoom), rotation (−15° to 15°), brightness enhancement, noise addition, and shifting (±15° horizontally and vertically). These transformations were applied multiple times to the same images to improve the model’s generalization capability. A total of 1745 images from 181 patients were used in the training and validation phases. To ensure class balance, equal-ratio data augmentation techniques were applied across all categories, expanding the dataset to 4189 images. The visuals related to dataset preparation are presented in Figure 1.

### 2.4. Study Design and Deep Learning

**Figure 2:**
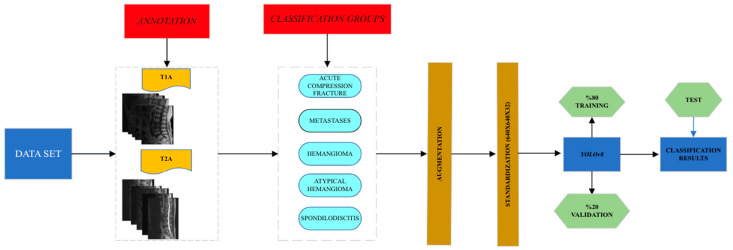
Workflow of the study.

In our study, images of five different pathologies—‘Acute Compression Fracture,’ ‘Spondylodiscitis,’ ‘Vertebral Atypical Hemangioma,’ ‘Vertebral Hemangioma,’ and ‘Vertebral Metastasis’—were used for model training. A total of 235 patients were included in the study. Of these, 63 had acute compression fractures, 69 had vertebral metastases, 41 had vertebral hemangiomas, 32 had vertebral atypical hemangiomas, and 30 were diagnosed with spondylodiscitis. The images were uploaded to the Roboflow platform, where the regions of interest were annotated with bounding boxes and labeled appropriately for classification. The dataset, consisting of 4179 images from 181 patients, was split into 80% for training and 20% for validation. The diagnostic distribution of the 181 patients used in the training and validation phases is as follows: 48 with acute compression fractures, 28 with hemangiomas, 20 with atypical hemangiomas, 25 with spondylodiscitis, and 60 with metastases. In addition, to optimally evaluate the model’s performance, an external test set comprising 218 images from 54 patients—acquired from the same MRI device and without any data augmentation—was used. Among the 54 patients in the external test set, 15 had acute compression fractures (15 lesions), 13 had hemangiomas (13 lesions), 10 had atypical hemangiomas (10 lesions), 7 had spondylodiscitis (12 lesions), and 9 had metastases (22 lesions). For deep learning, the YOLO-v8 architecture, an object detection algorithm, was employed. A workflow diagram illustrating the study process is presented in Figure 2.

#### YOLO-v8

The YOLO (You Only Look Once) algorithm is a fast method for detecting and recognizing objects in images in real-time [9]. First introduced in 2016 [9], the algorithm approaches object recognition as a regression problem. Using convolutional neural networks (CNNs), it simultaneously performs object detection and determines class probabilities in a single forward pass. The algorithm divides the image into a grid and encodes object information within each grid cell, ensuring efficient detection. YOLO predicts multiple bounding boxes for each grid cell. However, to compute the loss, only one box—the one with the highest Intersection over Union (IoU) with the ground truth—is selected as responsible for the object. Each bounding box estimates the object’s dimensions and aspect ratios. The loss calculation is based on the total squared error between predictions and ground truth. The loss function incorporates classification, localization, and confidence losses [9].

YOLO-v8, developed in 2023, is one of the latest versions of the YOLO framework [10]. The architecture of YOLO-v8 is optimized for fast and accurate object detection and consists of three main components:

The YOLO-v8 architecture consists of four primary components: a feature extractor, a feature fusion module, a prediction head, and a loss function. The feature extractor captures both low-level (edges, corners) and high-level (object shapes) representations by progressively downsampling the input image. The feature fusion module combines multi-scale features using structures like the Feature Pyramid Network (FPN) and Path Aggregation Network (PAN), enhancing the detection of lesions of varying sizes and signal characteristics.

The model’s prediction process generated bounding boxes, class labels, and segmentation masks by branching into dedicated pathways for classification, localization, and masking. The loss function is composed of cross-entropy loss (for classification and masking) and localization-specific losses (Distributed Focal Loss and Complete IoU Loss), which together optimize the model’s accuracy across all tasks [9].

**Figure 3:**
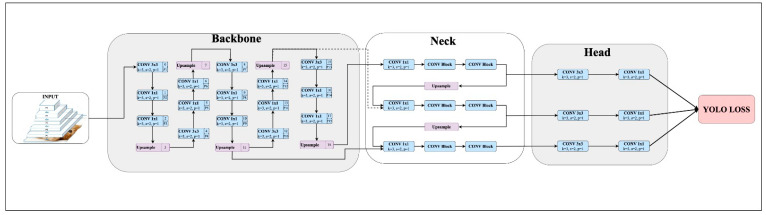
YOLO-v8 architecture.

The structure of YOLO-v8 integrates low-level details with high-level information, ensuring high accuracy and speed in multi-scale object detection. By increasing information density at each stage, the architecture is designed to detect objects of varying sizes effectively. The YOLO-v8 architecture is illustrated in Figure 3.

The YOLO algorithm is widely used in medical imaging analysis for detecting and classifying abnormalities such as fractures and tumors [9,11].

Additionally, YOLO has a wide range of applications, including autonomous vehicles (pedestrian and license plate detection), agriculture (plant disease analysis), security systems (face recognition, threat detection), unmanned aerial vehicles (terrain analysis, search and rescue), and industry (product defect detection) [10,11,12,13,14].

Currently, versions of YOLO exist from YOLO-v1 to YOLO-v12 [12,15]. The YOLO-v8 model used in this study demonstrates superior performance compared to previous versions (YOLO-v7, YOLO-v6, and YOLO-v5) based on test results from the COCO (Common Objects in Context) dataset, evaluated using two key performance metrics [16].

In this study, the YOLO-v8 model was trained using an NVIDIA RTX 4090 graphics processing unit (GPU) with CUDA functionality enabled. All processes were executed on the GPU using CUDA version 1.12.x, significantly reducing the training time. The training process utilized the Ultralytics 8.x, PyTorch 2.0, and OpenCV libraries. During model training, the batch size was set to 8, considering GPU memory limitations and ensuring that the model could learn effectively from a sufficient number of samples per iteration. The Adam optimizer was selected for its faster convergence and lower loss values compared to alternatives such as SGD or RMSProp, thanks to its adaptive learning rate and momentum-based updates.

The momentum value was set at 0.937, and the learning rate was dynamically adjusted using the YOLOv8x default scheduler throughout training. To balance the components of the loss function, the box and cls weights were set to 7.5 and 0.5, respectively. This helped improve the detection performance, particularly for smaller lesions.

The model’s sensitivity to these hyperparameters was monitored during testing, and it was observed that both batch size and optimizer choice had significant effects on the accuracy and convergence speed. Therefore, all settings were carefully tuned to achieve optimal performance on the dataset.

**Figure 4:**
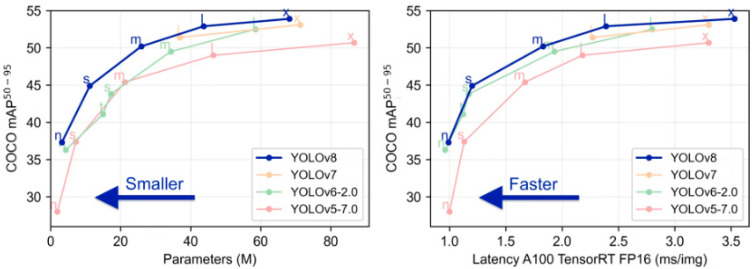
Comparison of YOLO-v8 and previous versions based on the COCO dataset graph [16].

According to the Ultralytics official documentation, YOLOv8 demonstrates a performance gain of approximately +1.5% to +2.2% in mean Average Precision (mAP) compared to its predecessor YOLOv5, depending on the model variant used (Figure 4). 

## 3. Results

**Figure 5:**
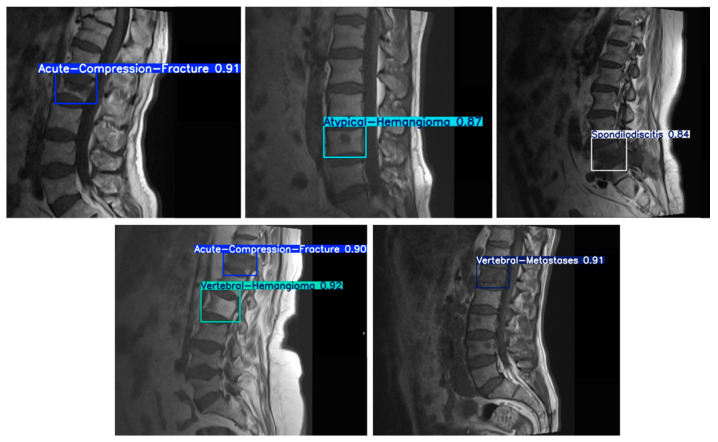
An example demonstrating the detection and classification of spinal lesions using bounding boxes with the YOLOv8 model. In the figure, each bounding box contains the detected lesion type (e.g., acute compression fracture, atypical hemangioma, and metastasis) along with the model’s confidence score for the prediction.

In our study, model performance metrics were determined based on analyses conducted separately for T1- and T2-weighted MRI sequences using the test dataset. The diagnostic groups defined for detection and classification are acute compression fracture, atypical hemangioma, vertebral hemangioma, metastasis, and spondylodiscitis (Figure 5)**.**

**Figure 6:**
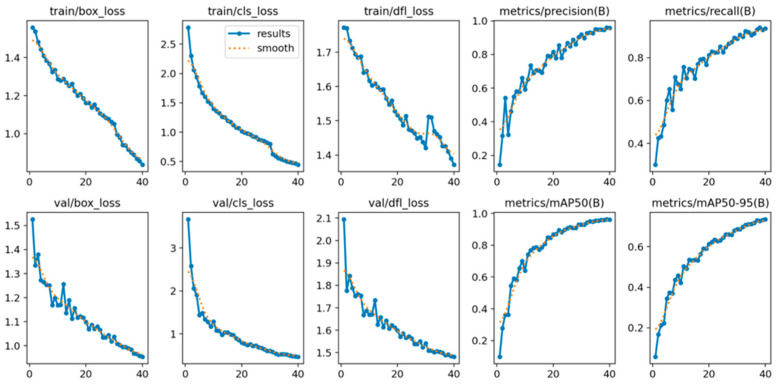
Graphs showing the loss values and validation metrics related to the performance training analysis of YOLO-v8.

Figure 6 shows the training results for the entire dataset. The training results are presented based on loss values. There are three types of loss information: box_loss, obj_loss, and cls_loss. Box_loss represents the loss indicating the difference between the predicted bounding boxes and the ground truth boxes. Obj_loss represents the difference in object presence for each grid. Cls_loss represents classification losses. A similar decreasing trend can be observed in these graphs. This indicates that the model is learning to accurately predict object classes. All loss values sufficiently decreased as the number of epochs increased, indicating that the training was successful. The fact that the precision, recall, mAP50, and mAP50-95 metrics increase and approach a value of 1 as training iterations continue suggests that the model is improving its ability to make accurate predictions. AP represents the average precision value calculated for each class, while mAP represents the overall mean average precision value calculated for all classes. mAP@0.5 corresponds to the mAP value calculated with an Intersection over Union (IoU) threshold of 0.5, whereas mAP@0.5:0.95 represents the mAP values calculated over an IoU range of 0.5 to 0.95 in 0.05 increments. The graph shows a continuous improvement in the mAP@0.5:0.95 metric, indicating that the model performs well across different object sizes. As a result, all findings confirm that the model’s training process was successfully conducted on the dataset using the default hyperparameters.

**Figure 7:**
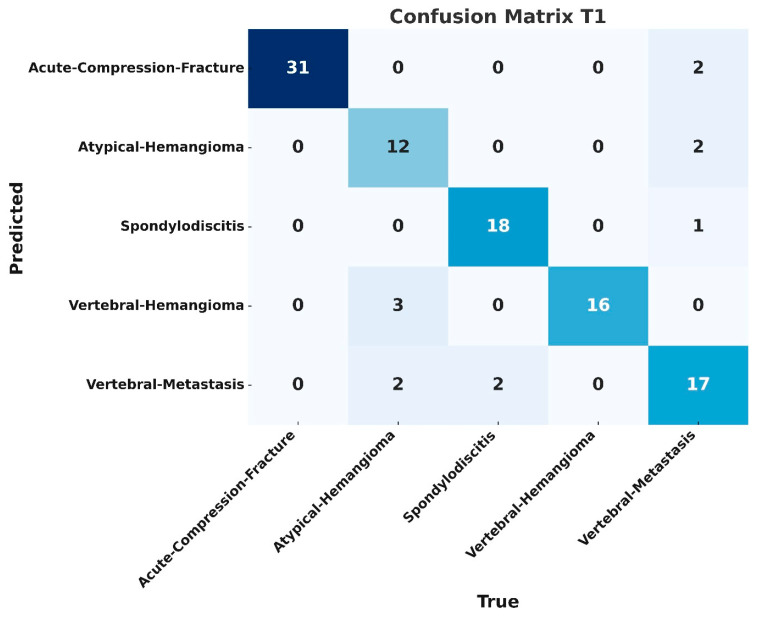
Classification accuracies based on the dataset obtained from T1-weighted images are as follows: 0.89 for acute compression fracture, 0.80 for metastasis, 0.73 for hemangioma, 0.84 for atypical hemangioma, and 0.94 for spondylodiscitis.

**Figure 8:**
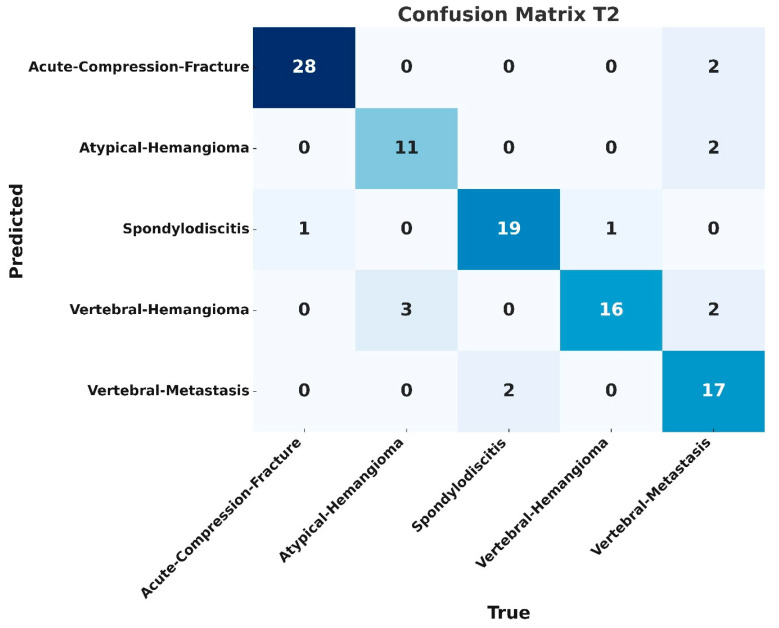
Classification accuracies based on the dataset obtained from T2-weighted images are as follows: 0.93 for acute compression fracture, 0.89 for metastasis, 0.73 for hemangioma, 0.79 for atypical hemangioma, and 0.86 for spondylodiscitis.

Although classification accuracy is not a directly appropriate metric for object detection models, it has been included here to enable comparison with results reported in previous studies. Based on correct detections with an Intersection over Union (IoU) ≥ 0.5, the overall classification accuracy was calculated as 0.84 for T1-weighted images and 0.85 for T2-weighted images. The confusion matrices for the datasets obtained with T1- and T2-weighted sequences are shown in Figure 7 and Figure 8.

Table 1 presents the performance metrics derived from the results of the external test set.

The F1 score is the harmonic mean of precision and recall metrics. In particular, it provides a better representation of model performance in imbalanced datasets. In our study, the F1 score–confidence curve graph shows an F1 score of 0.83 at a confidence value of 0.53 for the T1 dataset and 0.82 at a confidence value of 0.55 for the T2 dataset.

**Figure 9:**
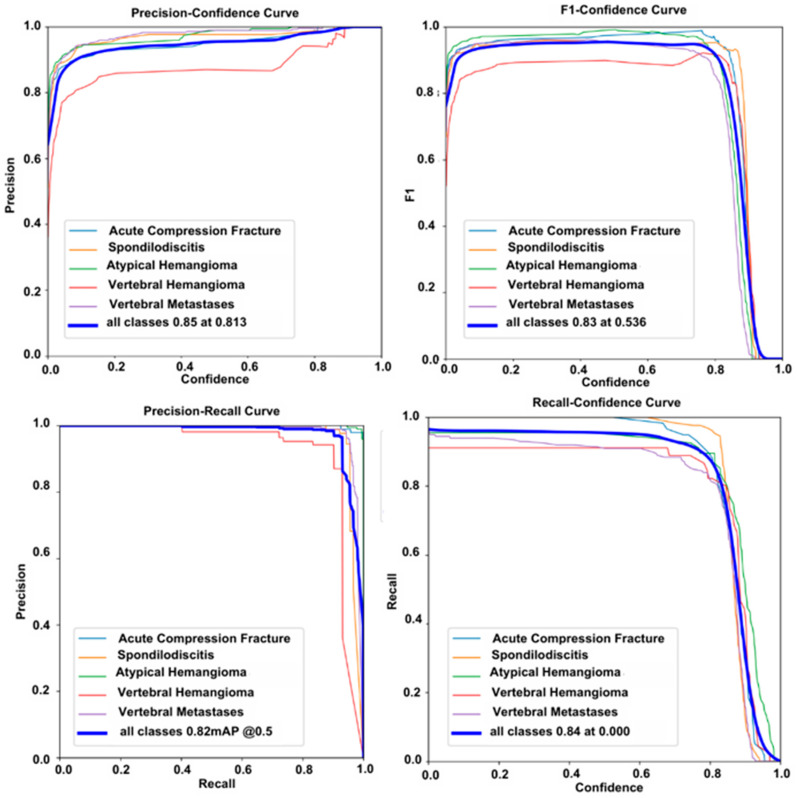
The Precision–Confidence curve, F1 Score–Confidence curve, Precision–Recall curve, and Recall–Confidence curve for the T1 dataset are shown.

**Figure 10:**
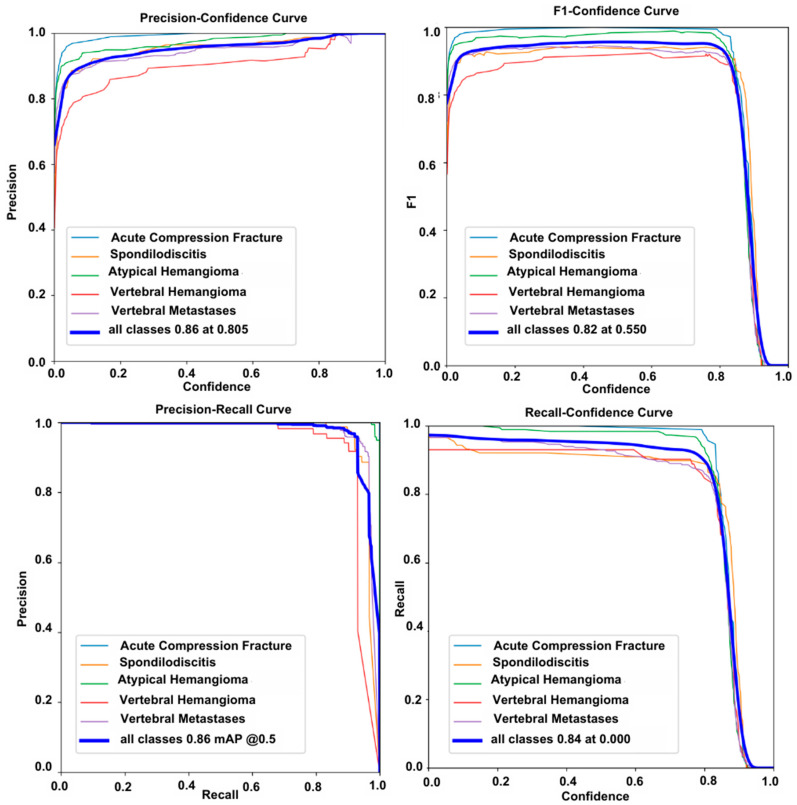
The Precision–Confidence curve, F1 Score–Confidence curve, Precision–Recall curve, and Recall–Confidence curve for the T2 dataset are shown.

The average precision (AP) score is calculated from the area under the Precision–Recall curve. The mAP score is the most effective performance metric for evaluating the accuracy of detection algorithms. In this study, at an IoU (Intersection over Union) value of 0.50, the mAP score obtained was 0.82 for the T1 dataset and 0.86 for the T2 dataset. All classification curve graphs are shown in Figure 9 for the T1 dataset and in Figure 10 for the T2 dataset.

For the performance on T1- and T2-weighted image datasets, the model achieved a precision of 0.85 and a recall of 0.82 for T1 images, while precision and recall for T2 images were 0.86 and 0.84, respectively.

## 4. Discussion

Spinal diseases are quite common. Apart from degenerative processes, benign and malignant lesions, compression fractures, and infectious diseases are frequently observed. MRI is the most sensitive imaging modality for diagnosis. However, the similar morphological characteristics of lesions pose a challenge in differential diagnosis. Pathologic tissue biopsy may be required for a definitive diagnosis. However, biopsy carries the risk of complications due to its invasive nature and requires expertise and experience to perform this procedure safely. Despite biopsy, diagnostic sensitivity is reported to be 85–90% for malignant diseases and 75–80% for benign diseases [17].

In this study, YOLO-v8 deep learning architecture, an object detection method, was used to automatically detect and classify metastases, hemangiomas, spondylodiscitis, and acute compression fractures, which are frequently seen in the vertebral corpus and have significant differences in diagnosis and treatment processes. The primary reason for selecting this architecture is its capability for real-time processing and low computational requirements. Considering that clinical environments often involve time and hardware limitations, YOLOv8 provides a practical alternative to conventional multi-stage architectures that focus solely on classification. Its ability to simultaneously perform detection and classification tasks in an efficient and integrated manner makes it particularly suitable for medical imaging applications. 

In the literature, artificial intelligence studies in medical imaging with YOLO and other object detection models have mainly focused on the detection of lesions or diseased regions [18]. In studies on the spine and its diseases, applications for the identification of spine regions, fracture detection, differentiation of benign and pathological fractures, tumor detection, and analysis of degenerative vertebral segments have come to the forefront, and very successful results have been demonstrated [18,19,20,21]. This study contributes uniquely to literature by being one of the first to simultaneously detect and classify both metastatic and benign vertebral lesions using MRI data through the YOLOv8 architecture. Its dual-task approach and application to multiple lesion types highlight its novelty and potential impact on the development of automated diagnostic systems in spinal imaging.

In addition to MRI, deep learning studies using CT and direct radiography are also available in literature. As an example of studies using CT, Koike et al. [22] used the YOLO-v5 algorithm for detection and the Inception-V3 algorithm for classification in their study to detect and classify vertebral segments and obtained very successful results. Studies using CT in areas such as the differentiation of benign and malignant vertebral fractures [23], segmentation of metastasis regions, detection of lytic and sclerotic metastases [24], and classification of spinal tuberculosis and acute compression fractures [25] have attracted attention with their high accuracy rates. When compared to the performance of surgeons and radiologists, these studies show similar or, in some cases, superior success rates.

In our study using MRI, T1- and T2-weighted sequence images were used, and the detection and classification processes were evaluated separately on datasets created from images obtained from these sequences. In the test set analyses, the F1 score was calculated as 0.83 for the T1-weighted sequence and 0.82 for the T2-weighted sequence, with a slightly higher performance for the T1 sequence. The mAP50, precision, and recall metrics were found to be 0.82, 0.85, and 0.82 for the T1 sequence, respectively. The same metrics were calculated as 0.86, 0.86, and 0.84 for the T2 sequence, respectively, and it was found that the T2 sequence outperformed the T1 sequence in terms of these metrics. These results show how different sequence types affect the diagnostic and classification performance of the model. In the confusion matrix evaluation, the most successful results for the T1 sequence were obtained in the spondylodiscitis and acute compression fracture groups with an accuracy of 0.94 and 0.89, respectively. For the T2 sequence, the highest accuracy values were obtained in the acute compression fracture group with 0.93 and in the metastasis group with 0.89. The lowest results for both sequences were obtained in the hemangioma group with an accuracy value of 0.73. The lower detection rate of hemangiomas is thought to be due to the smaller number of images used for training and testing compared to other lesion types. Additionally, variations in signal intensity within this class increase intra-class variability.

Deep learning studies on the differential diagnosis of spinal lesions have generally focused on the discrimination of benign and malignant vertebral fractures. For example, Liu et al. [26], using the TSCNN model, included 209 patients and obtained an accuracy of 0.95 for T1-weighted sequences and 0.90 for T2-weighted sequences. These results were superior to the classification performance of radiologists. Our study provides competitive results when compared to the work in this literature. The YOLO-v8 architecture we used is a single-stage object detection model that produces extremely fast results in the detection and classification of diseased regions. Considering this feature and classification performance, YOLO-v8 has the potential to provide a more practical and effective solution in clinical applications.

Using Faster R-CNN for detection and ResNetxt101 for classification, Liu et al. [27], aimed to differentiate between benign and malignant vertebral tumors in 585 patients. In this study, age information was also added as clinical data. An accuracy value of 0.82 was obtained, which was higher than the surgeons’ evaluations. The addition of age data increased the classification success. Our study provides more successful results than this study. In future studies, it is predicted that the differential diagnosis efficiency of deep learning models can be further improved by including clinical data in addition to imaging data. In different studies, successful results have been obtained for disc degeneration grading [28], spinal tuberculosis, and metastasis discrimination [29]. 

Models such as YOLOv12 have been developed recently, and the main focus of these models is to process image data faster and with a high capacity. However, in terms of detection and classification performance, they do not provide significant superiority compared to the model used in our study.

In addition, in our study, one-third of the images included in the metastasis group were lesions containing pathologic fractures. Metastatic lesions with and without pathological fractures are in the same group, and the distinction between these lesions and acute compression fractures is based on image attributes rather than fracture features. Most of the deep learning studies in this field in the literature have focused on the discrimination of benign and malignant fracture lesions. According to our results, our model seems to be successful in distinguishing between acute compression fractures and metastatic lesions with or without pathological fractures. This data constitutes one of the unique parts of our study, unlike other studies in the literature. 

Our study has some limitations. This study was performed retrospectively. Since the number of patients was relatively limited, image data augmentation was used to overcome this limitation. In addition, the diagnosis of the diseases was based only on imaging features, and clinical and imaging follow-up and histopathologic data were not included in this study. The datasets used in this study exhibit class imbalance in terms of the number of samples across lesion groups, which should be considered a significant limitation as it may lead to reduced model performance for the underrepresented classes. The model’s performance was not compared with alternative artificial intelligence models or expert evaluations (such as those of radiologists or clinicians), which constitutes a significant limitation of the study. Additionally, all imaging data used in this study were obtained from a single center using the same MRI device, and no external test data beyond the existing dataset were included in the analysis.

## 5. Conclusions

The analysis of multi-sequence MRI images using deep learning models for the differential diagnosis of vertebral corpus lesions presents an innovative approach that not only enhances diagnostic accuracy but also significantly improves the speed and efficiency of the process. The findings from this study demonstrate that deep learning algorithms have a high potential for success in medical imaging analysis and can serve as an effective complementary tool in diagnostic procedures. In this context, deep learning approaches are expected to make significant contributions to clinical applications by facilitating the accurate and rapid detection and differentiation of vertebral corpus lesions.

## Figures and Tables

**Table 1:** Test performance of the YOLOv8 model on the dataset.

Dataset	mAP	Precision	Recall	F1 Score	Accuracy
**T1**	0.82	0.85	0.82	0.83	0.84
**T2**	0.86	0.86	0.84	0.82	0.85

## Data Availability

The data presented in this study are available upon request from the corresponding author. The data are not publicly available due to privacy and ethical restrictions.
